# Electromagnetic Field (EMF) Radiation Alters Estrogen Release from the Pig Myometrium during the Peri-Implantation Period

**DOI:** 10.3390/ijms22062920

**Published:** 2021-03-13

**Authors:** Ewa Monika Drzewiecka, Wiktoria Kozlowska, Agata Zmijewska, Pawel Jozef Wydorski, Anita Franczak

**Affiliations:** Department of Animal Anatomy and Physiology, Faculty of Biology and Biotechnology, University of Warmia and Mazury in Olsztyn, Oczapowskiego 1A, 10-719 Olsztyn, Poland; kozlowskawiktoria@outlook.com (W.K.); agata.zmijewska@uwm.edu.pl (A.Z.); pawel.wydorski@uwm.edu.pl (P.J.W.)

**Keywords:** electromagnetic field, steroidogenesis, estrogens, aromatase, 17*β*HSD, uterus, myometrium, pigs

## Abstract

An electromagnetic field (EMF) may affect the functions of uterine tissues. This study hypothesized that EMF changes the estrogenic activity of pig myometrium during the peri-implantation period. Tissue was collected on days 15–16 of the gestation and incubated in the presence of EMF (50 and 120 Hz, 2 and 4 h). The *cytochrome P450 aromatase type 3* (*CYP19A3*) and *hydroxysteroid 17β dehydrogenase type 4* (*HSD17B4*) mRNA transcript abundance, cytochrome P450arom (aromatase), and 17*β* hydroxysteroid dehydrogenase 17*β*HSD) protein abundance and estrone (E_1_) and estradiol-17*β* (E_2_) release were examined using Real-Time PCR, Western blot and radioimmunoassay. Selected myometrial slices were treated with progesterone (P_4_) to determine whether it functions as a protector against EMF. *CYP19A3* mRNA transcript abundance in slices treated with EMF was less at 50 Hz (2 h) and greater at 120 Hz (2 and 4 h). *HSD17B4* mRNA transcript was greater in slices treated with EMF at 120 Hz (2 h). Progesterone diminished EMF-related effects on *CYP19A3* and *HSD17B4*. When P_4_ was added, EMF had suppressive (50 and 120 Hz, 2 h) or enhancing (50 Hz, 4 h) effects on aromatase abundance. The E_1_ release was lower after 4 h of EMF treatment at 50 Hz and P_4_ did not protect myometrial E_1_ release. In conclusion, EMF alters the synthesis and release of E_1_ and did not affect E_2_ release in the myometrium during the peri-implantation period.

## 1. Introduction

Estrogens are key regulators of female reproduction which affect corpus luteum (CL) lifespan, uterine receptivity, and are involved in embryo-maternal interactions [1,2,3,4,5]. Importantly, a pivotal source of estrogens in the uterus is the endometrium and the myometrium, in which steroidogenic activity depends on the reproductive status of the female [6,7,8]. Interestingly, past studies indicated that uterine steroidogenic activity might also be regulated not only by endocrine factors [9,10,11,12,13,14,15,16,17], but also by physical environmental factors, such as an electromagnetic field (EMF) [18,19,20].

The EMF at frequencies ranging from 50 to 120 Hz is generated by most electrical devices used in an everyday manner [21]. It has been well-documented that EMF at a frequency of 50 Hz and 120 Hz may cause DNA damage, induce structural, morphometric and physiological changes in testes, decrease the rate of fetal development, induce changes in brain protein conformation and generate alterations in signal transduction mechanisms involved in learning capacity and memory processes and induce anti-inflammatory responses [22,23,24,25,26]. In estrous-cyclic pigs, during the mid-luteal phase of the estrous cycle, an EMF at a frequency of 50 and 120 Hz increased myometrial estradiol-17*β* (E_2_) release within 4 h of treatment duration [18]. It was also determined that treatment with an EMF (50 Hz for 2 h, 120 Hz for 2 and 4 h) decreases the myometrial release of androstenedione (A_4_) [19]. Importantly, androgens are substrates for estrogen production [27]; thus, alterations in their production may cause a deregulation in estrogen production. The effect of an EMF on estrogen release from the myometrium has not yet been evaluated. Notably, the peri-implantation period is the most critical period for the success of female reproduction [28], during which the action of estrogens may determine the success of pregnancy [1,2,3,4,5].

Remarkably, in pigs during early pregnancy, estrogens act as anti-luteolytic factors, maintain luteoprotection and support progesterone (P_4_) production by CLs in early pregnancy [29,30,31]. It has become obvious that P_4_, which is a substrate for the steroidogenic pathway [8,27], is essential in the uterus to develop uterine receptivity and stimulates the transformation of uterine tissues to create an environment for embryonic development, implantation and placentation [32]. Recommendations are currently being considered for the prescription of P_4_ for gravid females to avoid early pregnancy complications and to increase endometrial receptivity to implantation [33,34]. Interestingly, P_4_ in estrous-cyclic females has functions that protect the pig uterine tissues against EMF radiation [18]. Nevertheless, in early pregnant females, P_4_ is not an obvious protection factor against EMF-related changes in uterine androgen production [19,20].

The present study aimed to determine the effect of an EMF (50 and 120 Hz, 8 militesla (mT), 2- and 4 h duration) treatment on (1) *cytochrome P450 aromatase type 3* (*CYP19A3*) and *hydroxysteroid 17β dehydrogenase type 4* (*HSD17B4*) mRNA transcript abundance, (2) cytochrome P450arom (aromatase) and 17*β* hydroxysteroid dehydrogenase (17*β*HSD) protein abundance, and (3) estrone (E_1_) and estradiol-17*β* (E_2_) release from the myometrium of pigs during the peri-implantation period (i.e., days 15 to 16 of gestation). To examine whether the inclusion of P_4_ may protect the myometrium against the EMF-related effects, selected myometrial slices were incubated in the presence of P_4_.

## 2. Results

### 2.1. EMF at 50 and 120 Hz Alter *CYP19A3* and *HSD17B4* mRNA Transcript Abundance in Myometrial Slices after 2 h and 4 h of In Vitro Incubation in the Presence or Absence of P_4_

The values for the main effects and interactions among factors affecting *CYP19A3* and *HSD17B4* mRNA transcript abundances, i.e., treatment with an EMF, duration of EMF treatment, and inclusion of P_4_ in culture medium are presented in Table 1. The relative abundance of *CYP19A3* and *HSD17B4* mRNA transcripts was affected by the interaction between an EMF treatment and the treatment duration (*p* = 0.03, and *p* = 0.0002, respectively). The relative abundance of *CYP19A3* mRNA transcript was also affected by the interaction between the treatment duration with the EMF and P_4_ inclusion (*p* = 0.0002) and by the interaction when an EMF treatment was imposed, the treatment duration of the EMF and P_4_ inclusion in the culture medium (*p* = 0.002).

**Figure 1:**
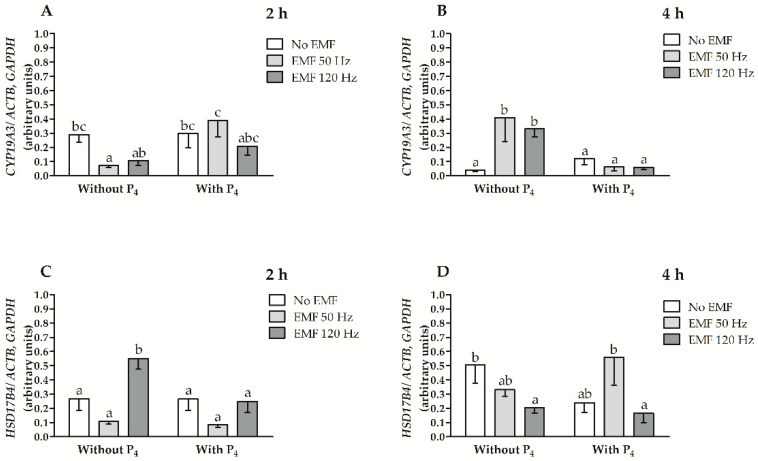
The relative abundance of *cytochrome P450arom* (*CYP19A3*) (**A**,**B**) and *17β hydroxysteroid dehydrogenase* (*HSD17B4*) (**C**,**D**) mRNA transcript abundance in myometrial slices collected from pigs during days 15–16 of early pregnancy and treated in vitro with EMF at 50 and 120 Hz for 2 h (**A**,**C**) and 4 h (**B**,**D**) of incubation with or without the inclusion of P_4_ in the culture medium. Data presented as mean ± SEM. Lower-case letters (a–c) above bars indicate statistically significant differences at *p* ≤ 0.05 (multi-way ANOVA).

After 2 h of treatment duration without the inclusion of P_4_ in the culture medium, the *CYP19A3* mRNA transcript abundance was lower in myometrial slices as a result of treatment with an EMF at a frequency of 50 Hz and was not altered in myometrial slices treated with an EMF at a frequency of 120 Hz (Figure 1A, *p* ≤ 0.05). After 2 h of treatment when there was the inclusion of P_4_ in the culture medium, the *CYP19A3* mRNA transcript abundance was not altered when treated with an EMF at a frequency of 50 and 120 Hz compared to slices not treated with an EMF (*p* > 0.05, Figure 1A). When treated with an EMF at a frequency of 50 Hz, the abundance of *CYP19A3* mRNA transcript was larger after 2 h of incubation both when there was the inclusion of P_4_ in the culture medium compared to myometrial slices and when there was no inclusion of P_4_ in the medium (*p* ≤ 0.05, Figure 1A). After 4 h of incubation without P_4_ inclusion in the medium, the abundance of *CYP19A3* mRNA transcript was higher when treated with an EMF at 50 Hz and 120 Hz, compared to myometrial slices not treated with an EMF (*p* ≤ 0.05, Figure 1B). In myometrial slices with P_4_ inclusion in the culture medium, after 4 h of incubation and imposing an EMF at 50 and 120 Hz, the *CYP19A3* mRNA transcript abundance was not altered (*p* > 0.05, Figure 1B). The abundance of *CYP19A3* mRNA transcript in myometrial slices after 4 h of incubation and the imposing of an EMF at 50 and 120 Hz was lower with the inclusion of P_4_ in the culture medium compared to myometrial slices incubated without P_4_ (*p* ≤ 0.05, Figure 1B).

The abundance of *HSD17B4* mRNA transcript was greater in myometrial slices after 2 h of incubation without the inclusion of P_4_ in the medium when treated with an EMF at the frequency of 120 Hz (*p* ≤ 0.05) and was not altered (*p* > 0.05) when there was treatment at 50 Hz (Figure 1C). In slices incubated with P_4_ inclusion in the culture medium after 2 h of incubation and imposing an EMF at 50 and 120 Hz, the abundance of *HSD17B4* mRNA transcript was not altered (*p* > 0.05, Figure 1C). The abundance of *HSD17B4* mRNA transcript in myometrial slices after 2 h of incubation without the inclusion of P_4_ in the culture medium and imposing an EMF at 120 Hz EMF was larger compared to slices incubated with the inclusion of P_4_ in the culture medium (*p* ≤ 0.05, Figure 1C). After 4 h of incubation without the inclusion of P_4_, the *HSD17B4* mRNA transcript abundance was lower in slices treated with an EMF at 120 Hz compared to slices not treated with an EMF (*p* ≤ 0.05, Figure 1D). When there was the inclusion of P_4_, the abundance of *HSD17B4* mRNA transcript was not altered in myometrial slices on which an EMF was imposed for 4 h at 50 and 120 Hz compared to slices not treated with EMF (*p* > 0.05, Figure 1D). The abundance of *HSD17B4* mRNA transcript was similar in myometrial slices both with and without the inclusion of P_4_ in the culture medium (*p* > 0.05, Figure 1D).

### 2.2. The Immunolocalization and the Abundance of Aromatase and 17*β*HSD in Myometrial Slices Treated with EMF at 50 and 120 Hz for 2 h and 4 h with or without the Inclusion of P_4_

**Figure 2:**
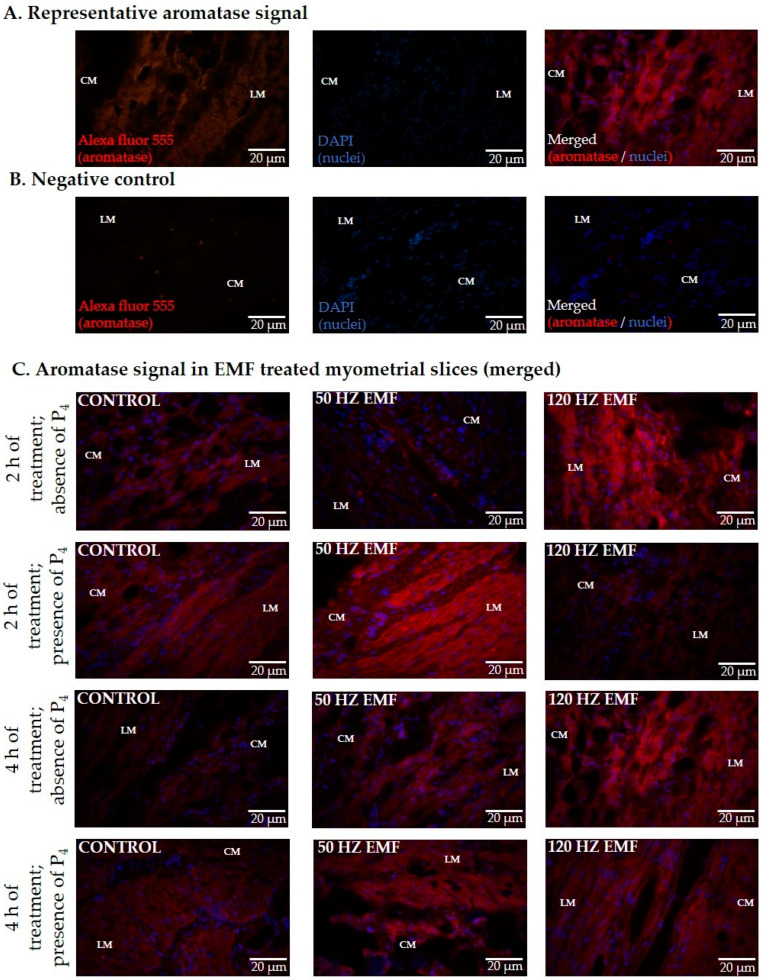
Randomly selected representative photographs of immunodetection of cytochrome P450arom (aromatase) in porcine myometrium of pigs during the peri-implantation period exposed in vitro to an electromagnetic field (EMF) at the frequency of 50 and 120 Hz, for 2 and 4 h in the presence or absence of P_4_. The panels show representative aromatase signal (**A**), negative control (**B**), and aromatase signal in EMF-treated myometrial slices (merged) (**C**). Magnification 400×. CM—circular muscle; LM—longitudinal muscle. The nuclei are stained with DAPI (blue) and binding sites are visualized with Alexa Fluor 555 (red).

**Figure 3:**
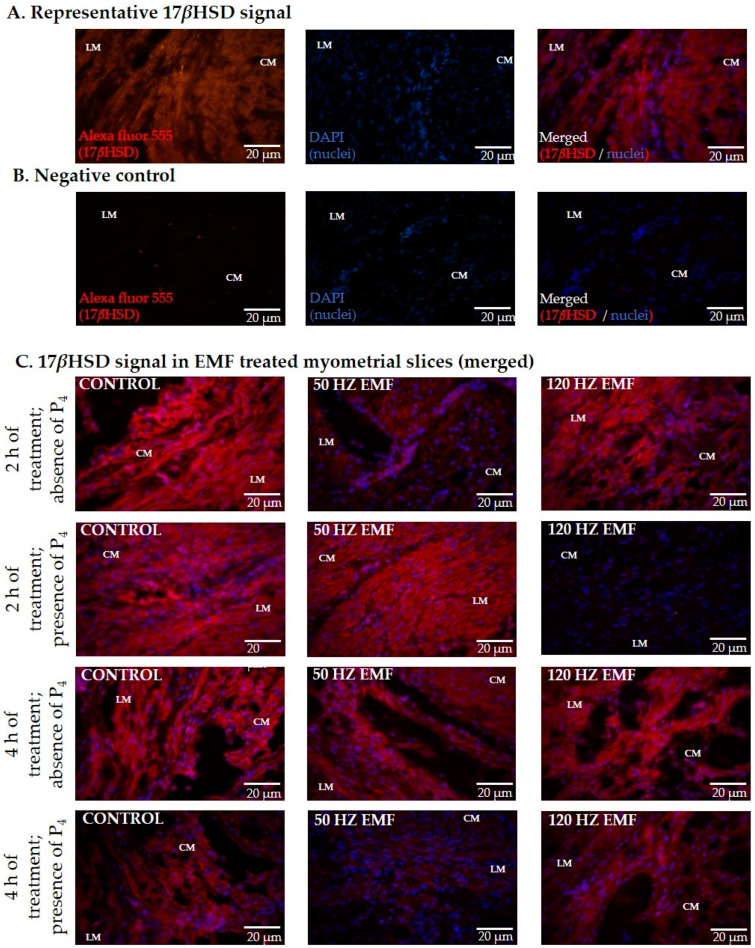
Randomly selected representative photographs of immunodetection of 17*β*-hydroxysteroid dehydrogenase (17*β*HSD) in porcine myometrium of pigs during the peri-implantation period exposed in vitro to an electromagnetic field (EMF) at 50 and 120 Hz for 2 and 4 h in the presence or absence of P_4_. The panels show representative 17*β*HSD signal (**A**), negative control (**B**), and 17*β*HSD signal in EMF-treated myometrial slices (merged) (**C**). Magnification 400×. CM—circular muscle; LM—longitudinal muscle. The nuclei are stained with DAPI (blue) and binding sites are visualized with Alexa Fluor 555 (red).

Aromatase and 17*β*HSD proteins were detected in the longitudinal (LM) and circular (CM) layers of the myometrium incubated for 2 and 4 h with or without treatment with an EMF and with or without the inclusion of P_4_ in the culture medium. Representative photographs visualizing aromatase and 17*β*HSD protein immunolocalization are presented in Figure 2 and Figure 3.

The values for the main effects and the interactions among factors affecting aromatase and 17*β*HSD protein abundances, i.e., treatment with an EMF, duration of EMF treatment, and inclusion of P_4_ in culture medium are presented in Table 2. Aromatase protein abundance was affected by treatment duration (*p* = 0.008), P_4_ inclusion (*p* = 0.005) and by the interaction between an EMF and the treatment duration (*p* = 0.00007). The relative abundance of 17*β*HSD protein was affected by an EMF (*p* = 0.004). Both aromatase and 17*β*HSD protein abundance were affected by an interaction that occurred when EMF was imposed and there was P_4_ inclusion in the culture medium (*p* = 0.01, and *p* = 0.001, respectively), and by interaction among treatments with an EMF, treatment duration and the P_4_ inclusion in the culture medium (both *p* = 0.007) (Table 2).

**Figure 4:**
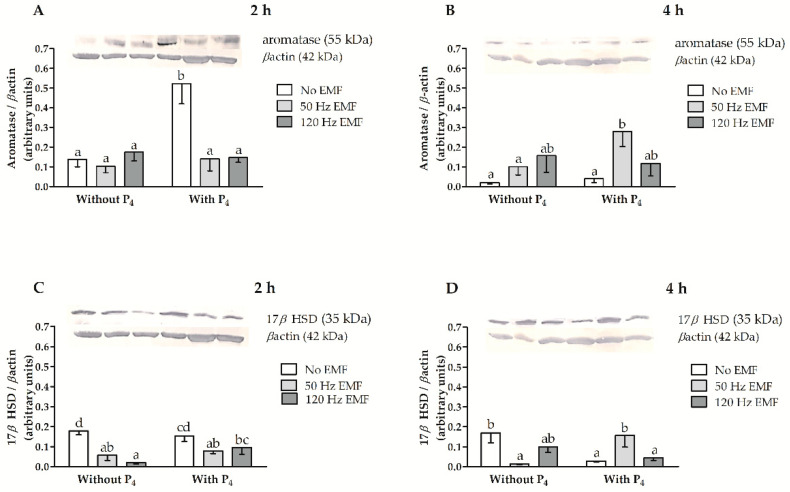
The relative abundance of cytochrome P450arom (aromatase) (**A**,**B**) and 17*β* hydroxysteroid dehydrogenase (17*β*HSD) (**C**,**D**) protein abundances in myometrial slices collected from pigs during days 15–16 of early pregnancy and treated in vitro with an EMF at 50 and 120 Hz for 2 h (**A**,**C**) and 4 h (**B**,**D**) of incubation with or without the inclusion of P_4_ in the culture medium. Data are presented as mean ± SEM. Lower-case letters (a–d) above bars indicate statistically significant differences at *p* ≤ 0.05 (multi-way ANOVA).

In myometrial slices without P_4_ inclusion in the culture medium after 2 h of incubation and imposing an EMF, the abundance of aromatase was similar to the control (*p* > 0.05, Figure 4A). In myometrial slices with P_4_ inclusion in the culture medium after 2 h of incubation and imposing an EMF at 50 and 120 Hz, the abundance of aromatase was less compared to slices not treated with an EMF (*p* ≤ 0.05, Figure 4A). In slices not treated with an EMF, the relative abundance of aromatase protein in myometrial slices after 2 h of incubation with P_4_ was larger compared with slices incubated without P_4_ (*p* ≤ 0.05, Figure 4A). When the EMF at 50 or 120 Hz was imposed, the abundance of aromatase protein was similar in myometrial slices incubated with and without P_4_ inclusion after 2 h of incubation (*p* > 0.05, Figure 4A). After 4 h of incubation without P_4_ in the culture medium the abundance of aromatase did not differ in myometrial slices treated or not with an EMF (*p* > 0.05, Figure 4B). In slices incubated with P_4,_ the abundance of aromatase after 4 h of incubation was larger when there was an EMF imposed at 50 Hz (*p* ≤ 0.05) and was not altered (*p* > 0.05) when there was an EMF imposed at 120 Hz (Figure 4B). In the myometrial slices treated with EMF at 50 Hz, the aromatase abundance was larger in slices incubated with P_4_ inclusion (*p* ≤ 0.05, Figure 4B) compared to slices incubated without P_4_ (*p* > 0.05, Figure 4B).

After 2 h of incubation without P_4,_ the abundance of 17*β*HSD protein was lower in myometrial slices when there was an EMF imposed at 50 and 120 Hz compared to untreated myometrial slices (*p* ≤ 0.05, Figure 4C). When there was the inclusion of P_4_, the abundance of 17*β*HSD was less when there was an EMF imposed at 50 Hz (*p* ≤ 0.05) and was similar (*p* > 0.05) when there was an EMF imposed at 120 Hz (Figure 4C). In slices treated with an EMF at 120 Hz, the abundance of 17*β*HSD protein was larger after 2 h of incubation with P_4_ compared to slices incubated without P_4_ (*p* ≤ 0.05, Figure 4C). After 4 h of incubation without P_4_ in the culture medium, the 17*β*HSD abundance was less when there was an EMF imposed at 50 Hz (*p* ≤ 0.05) and was similar in slices treated with an EMF at 120 Hz (*p* > 0.05, Figure 4D). When there was the inclusion of P_4_ in the culture medium, the abundance of 17*β*HSD was less when an EMF was not imposed, was larger in slices treated with an EMF at 50 Hz, and was similar in slices treated with an EMF at 120 Hz, compared to slices incubated without P_4_ (*p* ≤ 0.05, Figure 4B). Full-length blots are provided in Figure S1.

### 2.3. Effect of EMF at 50 and 120 Hz on E_1_ and E_2_ Secretion In Vitro from Myometrial Slices Incubated with or without the Inclusion of P_4_

The values for the main effects and the interactions among factors affecting E_1_ and E_2_ release, i.e., treatment with an EMF, duration of EMF treatment, and inclusion of P_4_ in culture medium are presented in Table 3. Estrone release was affected by treatment with an EMF (*p* = 0.00001), P_4_ inclusion (*p* < 0.0000001) and the interaction between EMF treatment and EMF treatment duration (*p* = 0.001). The release of E_2_ was affected by the P_4_ inclusion in the culture medium (*p* < 0.0000001) and the interaction between the treatment duration and the P_4_ inclusion (*p* = 0.02).

**Figure 5:**
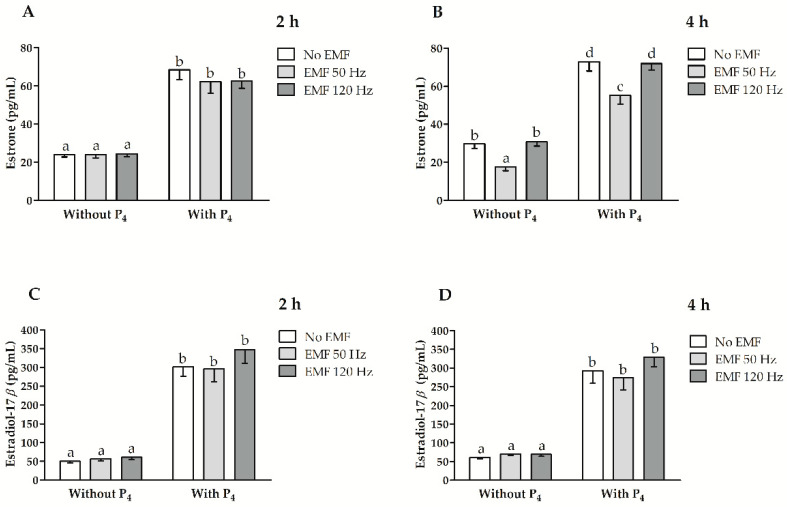
The myometrial release of estrone (**A**,**B**) and estradiol-17*β* (**C**,**D**) from myometrial slices collected from pigs during days 15–16 of early pregnancy and treated in vitro with an EMF at 50 and 120 Hz for 2 h (**A**,**C**) and 4 h (**B**,**D**) of incubation with or without the inclusion of P_4_ in the culture medium. Data is presented as mean ± SEM. Lower-case letters (a–d) above bars indicate statistically significant differences *p* ≤ 0.05 (multi-way ANOVA).

After 2 h of incubation, the myometrial E_1_ concentration in the culture medium did not differ for myometrial slices treated with an EMF at 50 and 120 Hz despite the inclusion of P_4_ in the culture medium (*p* > 0.05, Figure 5A). After 4 h of incubation with and without P_4_ inclusion in the culture medium, the concentration of E_1_ in the culture medium was less for myometrial slices when there was an EMF imposed at 50 Hz and was not altered when there was an EMF imposed at 120 Hz (*p* ≤ 0.05, Figure 5B). The concentration of E_2_ release was similar in the culture medium for slices treated with an EMF at 50 and 120 Hz after 2 and 4 h of incubation, despite the inclusion of P_4_ (*p* > 0.05, Figure 5C,D). When there was the inclusion of P_4_, the E_1_ and E_2_ concentration in the culture medium was larger than for slices incubated without the inclusion of P_4_, despite treatment with EMF and the treatment duration with an EMF (*p* ≤ 0.05, Figure 5A–D).

### 2.4. Estrogen Concentration in Uterine Flushings and Blood Plasma

**Figure 6:**
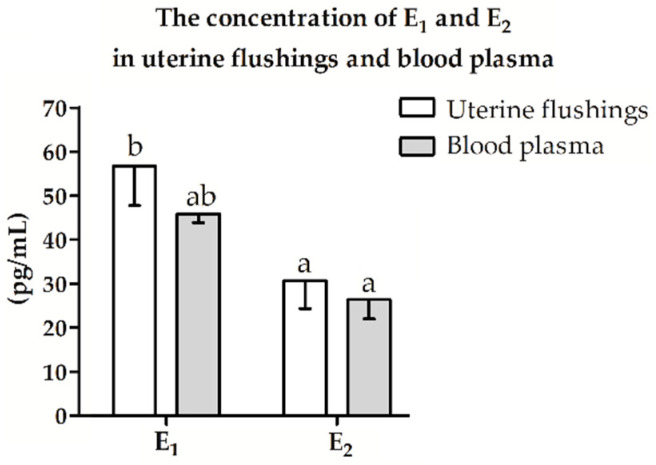
The concentration of estrone (E_1_) and estradiol-17*β* (E_2_) in uterine flushings and blood plasma of pigs during days 15–16 of early pregnancy. Data are presented as mean ± SEM. Lower-case letters (a, b) above bars indicate statistically significant differences *p* ≤ 0.05 (multi-way ANOVA).

The concentration of E_1_ and E_2_ in uterine flushings and blood plasma did not differ (*p* > 0.05, Figure 4). In the uterine flushings, the concentration of E_1_ was higher than the concentration of E_2_ (*p* ≤ 0.05, Figure 6). In blood plasma, the concentration of E_1_ and E_2_ did not differ (*p* > 0.05, Figure 6).

## 3. Discussion

The current study has provided, for the first time, evidence that an EMF treatment induces alterations in estrogen synthesis and release in the myometrium of pigs during the peri-implantation period. Specifically, the abundance of key enzymes involved in the production of estrogens, i.e., aromatase and 17*β*HSD, was altered in response to an EMF at the frequency of 50 and 120 Hz both on transcript and protein levels, but the effect of EMF treatment was variable due to the treatment duration of EMF, and/or P_4_ inclusion in the culture medium. Notably, EMF treatment affected the mRNA transcript and protein abundance for these steroidogenic enzymes, but the inductions were inconsistent between the mRNA transcript and protein levels. This observation was not surprising since the transcription and translation processes in Eukaryotes are separated in space and time [35]. Importantly, regardless of the P_4_ inclusion in the culture medium, myometrial E_1_ was less in slices treated with an EMF at a frequency of 50 Hz within a longer (4 h) treatment duration of EMF. None of the examined EMF frequencies or treatment durations of EMF affected E_2_ release from the myometrium.

The mechanism of steroid production is determined by cytochrome P450 17*α*-hydroxylase/C17-20 lyase (cytochrome P450c17), which is a rate-limiting enzyme controlling the entry of P_4_ and pregnenolone to the steroidogenic pathway [27,36]. Previously, it was documented that a longer (4 h) treatment duration of the EMF at a frequency of 50 Hz results in a greater cytochrome P450c17 protein abundance, but did not affect A_4_ release by the myometrium collected from pigs during the peri-implantation period [19]. The EMF radiation at a higher (i.e., 120 Hz) frequency led to an increased concentration of myometrial A_4_ when there was the inclusion of P_4_ in the culture medium within a longer (4 h) treatment duration of EMF, which coincided with the greater 3*β* hydroxysteroid dehydrogenase (3*β*HSD) abundance in the tissue [19]. The 3*β*HSD catalyzes 3*β*-hydroxysteroid dehydrogenation and Δ5 to Δ4 isomerization of pregnenolone and 17α-hydroxypregnenolone, dehydroepiandrosterone and androstenediol into progesterone and 17α-hydroxyprogesterone, A_4_ and testosterone (T), respectively [37]. Notably, A_4_ is the most potent and essential androgen in pigs that affects anabolic processes, morphogenesis, cellular proliferation and hyperplasia in the target tissue [38]. Androgens function as substrates for estrogen production in the reaction catalyzed by aromatase [39]. Therefore, the increased abundance of 3*β*HSD and greater concentration of A_4_ in the culture medium of myometrial slices incubated for longer treatment duration, when there was an EMF imposed, indicate the potential of an EMF to increase estrogen release in the tissue.

Interestingly, it was found that the basal myometrial abundance of *CYP19A3* mRNA transcript is decreased during the peri-implantation period when compared to its abundance in the myometrium collected from estrous-cyclic pigs, but the abundance of the encoded protein is mostly similar in gravid and non-gravid pig myometrium [10]. Thus, the myometrial competence to synthesize estrogens, measured as the abundance of aromatase, on days 15 to 16 of pregnancy and during the respective days of the estrous cycle is similar. The results of the current study indicated that myometrial abundance of *CYP19A3* mRNA transcript decreases in response to a relatively short (2 h) duration of EMF treatment at a frequency of 50 Hz, while it increases after a relatively long (4 h) duration of EMF treatment at a frequency of 50 and 120 Hz. Thus, the imposing of an EMF may destabilize the potential of the myometrium to synthesize estrogens. This study evaluated that the interaction among EMF treatment, duration of EMF treatment, and the inclusion of P_4_ to the culture medium affects the abundance of myometrial *CYP19A3* mRNA transcript. Moreover, it was evaluated that the inclusion of P_4_ to the culture medium diminished all the observed alterations in the abundance of *CYP19A3* mRNA transcript that occurred in the presence of EMF. The previous study indicated that the interaction between the EMF presence and the P_4_ inclusion into the culture medium also affects myometrial *CYP19A3* mRNA transcript abundance in pigs during the mid-luteal phase of the estrous cycle [18]. Nevertheless, it was evaluated that when there was an EMF imposed at 50 Hz, P_4_ functioned as a factor sensitizing myometrial *CYP19A3* mRNA transcript abundance to EMF treatment, whereas when there was an EMF imposed at 120 Hz, P_4_ functioned as a protective factor against EMF radiation [18]. Thus, the function of P_4_ in the modulation of myometrial *CYP19A3* mRNA transcript abundance in estrous-cyclic females depends on the frequency of EMF [18], whereas in early pregnant females P_4_ may function as a protective factor against alterations evoked by EMF treatment, despite the EMF frequency used (50 or 120 Hz) or the duration of treatment.

Notably, when the myometrium was treated with an EMF at 50 and 120 Hz for 2 or 4 h with no inclusion of P_4_ in the medium, the abundance of aromatase was not altered. In the presence of P_4_, the basal myometrial aromatase abundance after 2 h treatment duration was significantly greater than in slices incubated without P_4_ or with the inclusion of P_4_ and treated with an EMF at the frequency of 50 and 120 Hz. A significant increase in aromatase abundance in myometrial slices incubated in the medium supplemented with P_4_, when there was no EMF imposed, may indicate that the presence of a substrate for steroid hormone synthesis induces myometrial production of the enzyme required for estrogen synthesis. It is noteworthy that the previous studies indicated that in the myometrium of pigs during the peri-implantation period, the basal *CYP19A3* mRNA is decreased, whereas aromatase abundance is increased [10]. This phenomenon coincided with an increased level of P_4_ in uterine flushings [40]. Moreover, the results of studies performed on human primary cytotrophoblasts documented that a greater concentration of P_4_ in the culture medium corresponds to the increased abundance of aromatase in cytotrophoblasts cells [41]. These notions are in an agreement with the current results since the inclusion of P_4_ to the culture medium leads to a greater abundance of myometrial aromatase protein. Thus, the lack of increased abundance of aromatase in the myometrium treated by the EMF in the presence of P_4_ may indicate that EMF radiation may disturb the function of the P_4_ as a substrate for steroid hormone synthesis in the myometrium. Noteworthy, in studies using MCF-7 breast cancer cells, a co-treatment with P_4_ blocked cyclic adenosine monophosphate and interleukin 1*β* stimulated aromatase activity [42]. Thus, the relation between P_4_ presence and the abundance of aromatase might depend on tissue and cell type, but in porcine myometrium, the presence of P_4_ appears to generally increase aromatase abundance, but the EMF radiation may contribute to the disturbance of the relations between P_4_ and aromatase.

This study determined that a relatively longer treatment duration (4 h) of EMF leads to apparently different alterations in myometrial aromatase abundance. Specifically, there were no observed differences in basal aromatase abundance in myometrial slices incubated with P_4_ and not exposed to EMF, compared to slices incubated without P_4_ included in the culture medium. Interestingly, when EMF at a frequency of 50 Hz was imposed, the aromatase abundance was larger in myometrial tissue when P_4_ was also provided to the culture medium compared to slices incubated without P_4_. This phenomenon indicates that in pigs during the peri-implantation period, P_4_ functions as a factor that sensitizes translation processes in the myometrium to the EMF radiation, resulting in increased aromatase protein synthesis. It cannot be excluded that the increased concentration of aromatase protein could cause a suppression of *CYP19A3* mRNA expression. Such a correlation between RNA and protein expression profiles was previously documented in 23 human cell lines [43]. Although the presence of P_4_ in the culture medium apparently protected against EMF radiation, the transcriptional processes that resulted in the production of myometrial *CYP19A3* mRNA transcript, P_4_ also have the potential to sensitize translation processes to EMF radiation. These changes are likely to impact estrogen production in the myometrium of pigs during the peri-implantation period, potentially affecting CL lifespan, uterine receptivity and embryo-maternal interactions [1,2,3,4,5].

The current study determined that myometrial release of E_1_ in response to an EMF at 50 Hz (4 h) was lower despite the inclusion or not of P_4_ in the culture medium. Previously, it was found that in pigs during the peri-implantation period, the basal E_1_ release by the myometrium is similar to that which occurred in pigs during luteolysis [6]. Since the production of estrogens during early pregnancy in pigs is the result of androgen conversion [44] and A_4_ is converted to E_1_ [45], the observed lesser release of E_1_ in the response to EMF may indicate that the aromatization of A_4_ to E_1_ is disturbed as a consequence of EMF radiation at the frequency of 50 Hz. Importantly, this study found no clear relevance between the production of E_1_ and E_2_ and the expression levels for steroidogenic enzymes. This phenomenon can be a result of estrogen metabolism documented previously in ovary [46], but it cannot be excluded that it also occurs in uterine tissues. Further studies are needed to evaluate the effect of EMF treatment on estrogen metabolism in the myometrium. Notably, E_1_ is a less potent estrogen than E_2_, but, as confirmed within this study, E_1_ concentration in uterine flushings is higher than the concentration of E_2_. Taking into account the levels of estrogen production by myometrial slices, it cannot be excluded that the concentration of E_1_ in the uterine lumen is also the effect of the steroidogenic activity of the endometrium. Moreover, it should be noted that estrogens are considered as important regulators of uterine activity affecting embryo-maternal cross-talk during early pregnancy [47] and, especially E_2_, may exert a luteotrophic effect [48]. Notably, E_2_ is still considered as one of the embryonic signals for maternal recognition of pregnancy in pigs [5], but greater than the optimal concentration of E_2_ in uterine milieu during the peri-implantation period may induce cytotoxic effects in conceptuses [49]. Thus, any factor that could increase E_2_ production and disturb the slight balance of E_1_ and E_2_ in the uterus during the peri-implantation period may be considered as a potent disruptor of pregnancy in pigs.

As indicated above, EMF treatment at a frequency of 50 Hz significantly decreases in vitro production of E_1_ by myometrial tissue collected from pigs during the peri-implantation period despite the inclusion or not of P_4_ in the culture medium. Additionally, no EMF treatment-related effects were found on myometrial E_2_ production. Moreover, the inclusion of P_4_ does not have functions that protect the pig myometrium against the effect of EMF on E_1_ release. E_1_ might be converted to E_2_ via the activity of 17*β*HSD [27]. Results from the past study indicate that the expression level of 17*β*HSD in the endometrium of estrous-cyclic and pregnant pigs corresponds with the enzyme activity [50], and the myometrial activity of 17*β*HSD is lowered during the peri-implantation period when compared to the foregoing days of early pregnancy [17]. This phenomenon suggests that during the peri-implantation period, the potential of the myometrium to convert E_1_ to E_2_ is significantly lower, which may be recognized as a unique protective mechanism against the overproduction of E_2_ by the tissue. In this context, it is not surprising that EMF influences rather E_1_ than E_2_ release, despite the increased abundance of 3*β*HSD and the concentration of A_4_ in the culture medium of myometrial slices when there was an EMF imposed [19].

Regarding the results of the current study, the myometrial abundance of *HSD17B4* mRNA transcript and encoded protein in the presence of EMF and P_4_ is not stable and alters dynamically. Specifically, *HSD17B4* mRNA transcript abundance is greater in response to EMF treatment at a frequency of 120 Hz after 2 h of EMF treatment duration, but it is lower after 4 h of EMF treatment. The inclusion of P_4_ to the culture medium diminished the EMF-related effects on transcriptional processes. Furthermore, 17*β*HSD protein abundance decreased in response to EMF at the frequency of 50 Hz after short (2 h) and long (4 h) treatment duration and at a frequency of 120 Hz after short (2 h) treatment duration without the inclusion of P_4_ in the culture medium. Noteworthy, the observed increased abundance of *HSD17B4* mRNA transcript in myometrial slices exposed to EMF at a frequency of 120 Hz for 2 h is accompanied by low protein concentration. These facts may indicate that the phenomenon of RNA interference or changes in the level of post-translational modifications occurred [43,51]. However, these phenomena require further investigation. Interestingly, when there was an inclusion of P_4_ in the culture medium and EMF at 50 Hz was imposed, the myometrial 17*β*HSD protein abundance after relatively short (2 h) treatment duration decreased, but increased after a longer (4 h) duration of EMF treatment. Nevertheless, due to the already significantly lowered abundance of 17*β*HSD in the myometrium of pigs during the peri-implantation period [17], these changes did not affect the final E_2_ production despite the various parameters of EMF treatment used within this study. Therefore, it seems that EMF influences the synthesis of estrogens in the myometrium mostly at the level of transcription and translation and influences the E_2_ synthesis to a lesser extent.

## 4. Materials and Methods

### 4.1. Animals and Collection of Myometrial Tissue

Post-pubertal pigs (*n* = 5, Sus scrofa f. domestica, Polish Landrace × Great White Polish, aged 10 months, weighing 95–110 kg) were observed for estrus behavior in the presence of an intact boar. Pigs were naturally bred twice, i.e., on the first and the second day of the second estrus. The second mating was assigned as the first day of pregnancy. On days 15 to 16 of pregnancy, pigs were slaughtered in the local abattoir using standard procedures (Rozdroże, Poland), provided in the professional slaughterhouse, using humane procedures. All living pigs were handled by the authors. After slaughter, the entire uteri were excised, placed in ice-cold sterile phosphate-buffered saline (PBS, pH = 7.4) supplemented with 3% antibiotic–antimycotic solution (Sigma Aldrich, St Louis, MO, USA). Whole blood was collected into heparinized vials during slaughter. Uteri and blood were transported within 30 min to the Laboratory of Animal Anatomy and Physiology, Department of Biology and Biotechnology, University of Warmia and Mazury in Olsztyn, Olsztyn, Poland, ensuring cooling conditions (4 °C). The stage of pregnancy was confirmed by the morphology of the ovaries and CLs quality [52,53] and the presence and morphology of conceptuses flushed from the uterine horns with sterile saline [54,55]. In the laboratory, blood samples were centrifuged (7500× *g*, 10 min, 4 °C), and blood plasma was stored at −20 °C for further E_1_ and E_2_ concentration analyses. The uteri were flushed with 20 mL of sterile ice-cold phosphate-buffered saline (PBS, pH = 7.4) supplemented with 3% antibiotic—antimycotic solution (Sigma Aldrich, St Louis, MO, USA). Uterine flushings were collected for further determination of E_1_ and E_2_ concentrations. Uterine horns were opened longitudinally, the perimetrium was discarded by careful scraping and the myometrial fragments were picked by tweezers. Individual myometrial fragments were washed in an ice-cold PBS (pH = 7.4) supplemented with 3% of the antibiotic–antimycotic solution (Sigma Aldrich, St Louis, MO, USA) and standardized to 95–105 mg-weighting, 2–3-mm-thick fragments used for further in vitro incubation. All myometrial fragments collected from one pig were considered one biological repeat (*n*).

### 4.2. In Vitro Incubation and EMF Treatment System

Prepared standardized fragments of the myometrium were placed in 24-well culture dishes and covered with 1 mL of the pre-incubation medium (M199, Sigma Aldrich, St Louis, MO, USA; 0.1% BSA, Carl Roth GmBH + Co KG, Mühlburg, Karlsruhe, Germany; 1% antibiotic–antimycotic solution, Sigma Aldrich, St Louis, MO, USA) and incubated in an EMF exposure system as previously described [19]. Briefly, myometrial tissue slices were first preincubated for 2 h at 37 °C, 95% O_2_ and 5% CO_2_ in a water-shaking bath and then incubated in fresh medium (the same composition) or fresh medium supplemented with P_4_ (10^−5^ M, SERVA Electrophoresis GmbH, Heidelberg, Germany). The EMF was generated by a Magneris apparatus (Astar, Bielsko-Biala, Poland), ensuring exposition to sinusoidal EMF at a frequency of 50 or 120 Hz and magnetic induction of 8 mT for 2 and 4 h of in vitro incubation. Control fragments of myometrial tissue were not treated with an EMF. The prevention of fields overlapping fields was ensured by a distance (50 cm) separation between water baths in which incubation was held. The rationale of the used EMF parameters (50 and 120 Hz, 8 mT, 2, and 4 h of treatment) has been described previously [18,56]. Moreover, EMF at frequencies of 50 and 120 Hz is classified as an extremely low electromagnetic field, which is common in the environment of living organisms [21], and a magnetic induction at 8 mT is a typical exposure level of the magnetic induction for devices and equipment used for therapeutic purposes or in industry [57]. The graphical presentation of the EMF exposure system was presented previously by Franczak et al. (2020). During the in vitro incubation, the thermal conditions were monitored to exclude the bias of the temperature on the examined parameters. After in vitro incubation, tissue fragments were collected and snap-frozen in liquid nitrogen (−196 °C) and stored at −80 °C for further analysis of mRNA transcript and protein abundances. The samples of the culture medium were collected and stored at −20 °C for further determination of E_1_ and E_2_ concentrations.

### 4.3. Determination of *CYP19A3* and *HSD17B4* mRNA Transcript Abundance

Total RNA was extracted from myometrial tissue using TRI-Reagent (Sigma Aldrich, Germany) following the standard protocol. The details of RNA extraction and preparation for further analyses were described previously [19]. RNA precipitates were used for analyses that were integral electrophoretically (separation in 1.5% agarose gel) and possessed an optical density ration A260/A280 in the range of 1.8–2.0 (spectrophotometer Tecan, Männedorf, Switzerland). The Real-Time PCR was performed according to the guidelines provided by Bustin et al. (2009) [58]. The amplification was conducted using 4 pg/μL aliquots of extracted RNA, TaqMan^®^RNA-to- 1-Step Kit (Applied Biosystems, Foster City, CA, USA) and specific TaqMan probes provided by Applied Biosystems (Foster City, CA, USA) listed in Table 4. Amplification was conducted in an AriaMX apparatus (Agilent Technologies, Santa Clara, CA, USA), with a standard thermal profile suggested by the producer. The Ct values were obtained with Aria 1.6 software (Agilent Technologies, Santa Clara, CA, USA) and used for cycle threshold (Ct) calculation. The Ct values of the tested genes were normalized with the geometrical mean of the reference genes and used for 2^−∆∆Ct^ calculation [59].

### 4.4. Immunodetection of Aromatase and 17*β*HSD

The immunodetection of aromatase and 17*β*HSD was performed using immunofluorescence. Firstly, 6-μm-thick cryosections of myometrial tissue were fixed in a 4% paraformaldehyde (P.P.H. STANLAB, Lublin, Poland) for 15 min (room temperature, RT) and subsequently washed 3 × 10 min with Tris-buffered saline containing Tween 20 (TBS-T buffer: 10 mM Tris, 150 mM NaCl, 0.1% Tween 20). To prevent non-specific labeling, the slides were then covered with a blocking buffer containing 20% of a normal donkey serum (EMD Millipore, Billerica, MA, USA) and 0.1% of Triton X-100 (Sigma Aldrich, St Louis, MO, USA) in PBS for 1 h at 4 °C. Next, the slides were washed 3 × 10 min with TBS-T and covered with the primary antibodies rabbit anti-cytochrome P450arom and rabbit anti-17*β*HSD diluted in TBS-T for 18 h at 4 °C, listed in Table 5. Slices assigned for negative control were covered with TBS-T without primary antibodies. Next, slides were washed 3 × 10 min with TBS-T (RT), covered with donkey anti-rabbit secondary antibodies Alexa Fluor 555 (A32794, Life Technologies, Eugene, OR, USA) diluted in PBS (1:1500) and incubated at 4 °C for 1 h in darkness to prevent photobleaching. Subsequently, the slides were washed in PBS (3 × 5 min) and mounted with Fluoroshield with 4′,6-diamidino-2-phenylindole (DAPI, Sigma Aldrich, St Louis, MO, USA) for further analysis with an epifluorescent BX 51 microscope (Olympus, Tokyo, Japan) under 400-fold magnification. The images were archived with a type DP72 digital camera (Olympus, Tokyo, Japan).

### 4.5. Determination of Aromatase and 17*β*HSD Protein Abundance

Total protein was extracted from myometrial tissue as described previously [19]. The 15-µg aliquots of the protein extracts were adjusted to a total volume of 32 µL and mixed with 8 µL of loading buffer containing 6 × Laemmli sample buffer (0.35 M Tris-HCl, pH = 6.8, SDS 10%, glycerol 30%, Dithiothreitol (DTT) 0.6M, bromophenol blue 0.175 M) and 1 M DTT, used in a 1:1 proportion. Protein ladder (PageRuler™ Plus, #26619. Thermo Fisher Scientific, Waltham, MA, USA) was diluted 4 × in water and mixed with the same loading buffer in a 1:5 proportion. Protein aliquots and protein ladder were denatured at 99 °C for 3 min and immediately used for SDS-PAGE electrophoresis (PowerEase 90 W, Life Technologies, Eugene, OR, USA). Samples were concentrated in a 4% stacking gel (15 mA/gel and 40 mV/gel) and then resolved in a 10% running gel (25 mA/gel, 80 mV/gel) in 1 × Tris-glycine-SDS buffer (T7777, Sigma Aldrich, St Louis, MO, USA). The samples were then transferred to nitrocellulose membranes (0.45 µm using a semi-dry transblot apparatus (constant 25 V, 2.4 mA, 12 min, Pierce Power Blot Cassette, Thermo Scientific, Waltham, MA, USA). After transfer, membranes assigned to aromatase and 17*β*HSD abundance analyses were blocked for 2 h at 4 °C in TBS-T containing 1% of bovine serum albumin fraction V (BSA, Carl Roth GmbH + Co KG, Mühlburg, Karlsruhe, Germany) and membranes assigned to *β*-actin abundance analysis were blocked for 2 h at RT in TBS-T buffer containing 5% of BSA. After blocking, the membranes were washed for 3 × 10 min in TBS-T buffer and then incubated overnight at 4 °C with primary antibodies (Table 5). Next, the membranes were washed 3 × 5 min in TBS-T buffer and incubated with secondary alkaline phosphatase-conjugated goat anti-rabbit antibodies (sc-2057, Santa Cruz Biotechnology, Dallas, TX, USA) used at a concentration of 0.2 μg/mL for 2 h (for *β*-actin) or 1.5 h (for aromatase and 17*β*HSD) at 4 °C or RT, respectively. Subsequently, membranes were washed 3 × 5 min in TBS-T buffer and the immunoreactive bands were visualized using a staining solution containing 2% of NBT/BCIP stock solution in 0.1 M Tris-HCl, pH = 9.5 (20 °C), 0.1 M NaCl, 0.05 M MgCl2 (Merck, Kenilworth, NJ, USA) in darkness for 30 s (for *β*-actin), 90 s (for aromatase) or 60 s (for 17*β*HSD). After visualization, the reaction was stopped in deionized water containing 0.5 M of ethylenediaminetetraacetic acid. The blots were subsequently evaluated for further calculations of the optical density of the bands using ImageJ open-source software. The optical density of the detected protein bands was normalized with the optical density of *β*-actin bands.

### 4.6. Determination of E_1_ and E_2_ Concentrations in Blood Plasma, Uterine Flushings and the Culture Medium

The concentrations of estrogens were determined using radioimmunoassay [60]. The cross-reactivity of antisera against E_1_ and E_2_ have been reported previously [61]. The extraction efficiencies of E_1_ and E_2_ were 88.55% and 86.55%, respectively. The assay sensitivity was 1 pg and the inter- and intra-assay coefficients of variation were 5.08% and 3.74%, respectively, for E_1_ and 6.70% and 3.56% for E_2_, respectively.

### 4.7. Statistical Analysis and Data Presentation

The results are presented as the mean ± SEM. The differences in mRNA transcript abundance were analyzed using 2^−∆∆Ct^ values. The abundance of proteins was analyzed using mean values of the optical density of detected protein bands normalized with the optical density of *β*-actin bands values. Each value was then log-transformed for hormone concentration analyses. All data were analyzed using a multi-way analysis of variance (ANOVA) and Fisher’s least significant difference (LSD) post hoc test, with the main factors as follows: 1/treatment with an EMF, which stands for control (no treatment with an EMF) and treatment with an EMF at 50 or 120 Hz; 2/duration of EMF treatment, which stands for 2 and 4 h treatment duration; 3/the inclusion of P_4_ in medium, which means the inclusion or no inclusion of P_4_ in the culture medium. The results obtained within 2 and 4 h of incubation were then considered separately and once again analyzed using multi-way ANOVA and Fisher’s LSD post-hoc tests. Statistically significant differences were considered at *p* ≤ 0.05.

## 5. Conclusions

In the myometrial tissue of pigs during the peri-implantation period, EMF treatment affects the potential for the synthesis of estrogens. The consequences of EMF radiation in the myometrium may depend on the basal tissue potential for the production of estrogens. The inclusion of P_4_ in the culture medium diminishes most of the observed EMF treatment-related effects on the level of transcriptional processes that result in the production of mRNA transcripts for these steroidogenic enzymes in the myometrium of pigs. On the contrary, on the level of translation processes that result in protein synthesis, P_4_ may sensitize myometrium to EMF radiation. Importantly, EMF at a low frequency of 50 Hz decreases myometrial E_1_ release after a relatively long (4 h) treatment duration, with or without the P_4_ inclusion and does not alter E_2_ release. In the pig myometrium, EMF leads to lowered E_1_ release. Thus, this study provides evidence that EMF can be recognized as a potent disruptor of steroidogenesis in the uterus of females during the peri-implantation period.

## Figures and Tables

**Table 1:** The main factors and the interactions affecting the *cytochrome P450arom* (*CYP19A3*) and *17β hydroxysteroid dehydrogenase* (*HSD17B4*) mRNA transcript abundance in porcine myometrium collected from pigs during the peri-implantation period (days 15–16 of pregnancy) and treated in vitro with an EMF at 50 and 120 Hz for 2 and 4 h of incubation with or without the inclusion of P_4_ (multi-way ANOVA).

	*CYP19A3* mRNA		*HSD17B4* mRNA	
Factor	F	*p*	F	*p*
Treatment with an EMF ^1^	0.64626	0.530705	0.2970	0.744938
Duration of EMF treatment ^2^	1.78356	0.191135	2.5835	0.117231
Inclusion of P_4_ in medium ^3^	0.15396	0.697379	1.9135	0.175597
Treatment with an EMF × Duration of EMF treatment	4.01165	0.027886	11.1303	0.000191
Treatment with an EMF × The inclusion of P_4_ in medium	0.89975	0.416711	3.0523	0.060373
Duration of EMF treatment × The inclusion of P_4_ in medium	16.93924	0.000253	0.7142	0.403948
Treatment with an EMF × Duration of EMF treatment × The inclusion of P_4_ in medium	7.47019	0.002176	3.1239	0.056823

^1^ Control (no treatment with an EMF) or treatment with an EMF at 50 or 120 Hz; ^2^ Treatment with an EMF for 2 or 4 h of incubation; ^3^ The presence or absence of P_4_ in the culture medium (10^−5^ M).

**Table 2:** The main factors and the interactions affecting the aromatase and 17*β*HSD protein abundance in porcine myometrium collected from pigs during the peri-implantation period (days 15–16 of pregnancy) and treated in vitro with an EMF at 50 and 120 Hz for 2 and 4 h of incubation with or without the inclusion of P_4_ (multi-way ANOVA).

	Aromatase		17*β*HSD	
Factor	F	*p*	F	*p*
Treatment with an EMF ^1^	0.3757	0.689390	6.2235	0.004293
Duration of EMF treatment ^2^	7.7595	0.008376	0.4887	0.488369
Inclusion of P_4_ in medium ^3^	9.1335	0.004537	0.0361	0.850231
Treatment with an EMF × Duration of EMF treatment	12.5524	0.000069	2.7590	0.074853
Treatment with an EMF × The inclusion of P_4_ in medium	4.8927	0.013022	7.7074	0.001408
Duration of EMF treatment × The inclusion of P_4_ in medium	1.6380	0.208562	1.5019	0.227210
Treatment with an EMF × Duration of EMF treatment × The inclusion of P_4_ in medium	5.7070	0.006915	5.6520	0.006703

^1^ Control (no treatment with an EMF) or treatment with an EMF at 50 or 120 Hz; ^2^ Treatment with an EMF for 2 or 4 h of incubation; ^3^ The presence or absence of P_4_ in the culture medium (10^−5^ M).

**Table 3:** The main factors and the interactions affecting the concentration of estrone and estradiol-17*β* in the culture medium, released by porcine myometrium collected from pigs during the peri-implantation period (days 15–16 of pregnancy) and treated in vitro with an EMF at 50 and 120 Hz for 2 and 4 h of incubation with or without the inclusion of P_4_ (multi-way ANOVA).

	Estrone		Estradiol-17*β*	
Factor	F	*p*	F	*p*
Treatment with an EMF ^1^	12.20	0.000012	2.09	0.127233
Duration of EMF treatment ^2^	0.78	0.378135	2.30	0.131376
Inclusion of P_4_ in medium ^3^	453.97	0.000000	773.30	0.000000
Treatment with an EMF × Duration of EMF treatment	6.75	0.001536	0.05	0.955168
Treatment with an EMF × The inclusion of P_4_ in medium	0.64	0.526061	1.26	0.287898
Duration of EMF treatment × The inclusion of P_4_ in medium	0.10	0.755648	5.63	0.019079
Treatment with an EMF × Duration of EMF treatment × The inclusion of P_4_ in medium	1.44	0.239492	0.22	0.806063

^1.^ Control (no treatment with an EMF) or treatment with an EMF at 50 or 120 Hz; ^2.^ Treatment with an EMF for 2 or 4 h of incubation; ^3^ The presence or absence of P_4_ in the culture medium (10^−5^ M).

**Table 4:** Taq Man probes used for determination of *cytochrome P450arom* (*CYP19A3*) and *17β-hydroxysteroid dehydrogenase* (*HSD17B4*) mRNA transcript abundance in the myometrium of pigs during the peri-implantation period treated in vitro with an electromagnetic field at 50 and 120 Hz for 2 and 4 h in the presence or absence of P_4_.

Target Gene Symbol	Target Gene Name	Taq Man Assay IDs
*CYP19A3*	cytochrome P450arom	Ss03384905_uH
*HSD17B4*	17*β*-hydroxysteroid dehydrogenase	Ss04245958_g1
Reference genes		
*ACTB*	*β*-actin	Ss03376081_u1
*GAPDH*	glyceraldehyde 3-phosphate dehydrogenase	Ss03374854_g1

**Table 5:** The primary antibodies used for immunofluorescence and Western blotting analyses.

Name	Type	Catalog No./Company	Host	Concentration
Anti-*β*-actin	Primary	A2066 (Sigma Aldrich)	Rabbit	2 μg/mL
Anti-aromatase	Primary	A7981 (Sigma Aldrich)	Rabbit	1 μg/mL
Anti-17*β*HSD	Primary	Orb137855 (Biorbyt)	Rabbit	1 μg/mL

## Data Availability

All of the data are presented in the study. The raw data used for the preparation of the presented results are available on request from the corresponding author.

## Supplementary Materials

The following are available online at https://www.mdpi.com/1422-0067/22/6/2920/s1. Figure S1: Representative full-length Western blot bands showing aromatase, 17*β*HSD and *β*-actin proteins in porcine myometrial explants exposed to an electromagnetic field (EMF) at 0, 50 and 120 Hz for 2 or 4 h in the presence or absence of P_4_.
