# Supplementary material for: Electromagnetic Field (EMF) Radiation Alters Estrogen Release from the Pig Myometrium during the Peri-Implantation Period
Source: Int J Mol Sci. 2021 Mar 13;22(6):2920. doi: 10.3390/ijms22062920 (PMC7999543; doi:10.3390/ijms22062920)
Supplement: Supplementary file 1 [file ijms-22-02920-s001.pdf]

Electromagnetic Field (EMF) Radiation Alters Estrogen Release from the Pig Myometrium during the Peri-Implantation Period

Ewa Monika Drzewiecka, Wiktoria Kozlowska, Agata Zmijewska, Pawel Jozef Wydorski, Anita Franczak

Figure S1: Representative full-length Western blot bands showing aromatase, 17*β*HSD and *β*-actin proteins in porcine myometrial explants exposed to an electromagnetic field (EMF) at 0, 50 and 120 Hz for 2 or 4 h in the presence or absence of P_4.

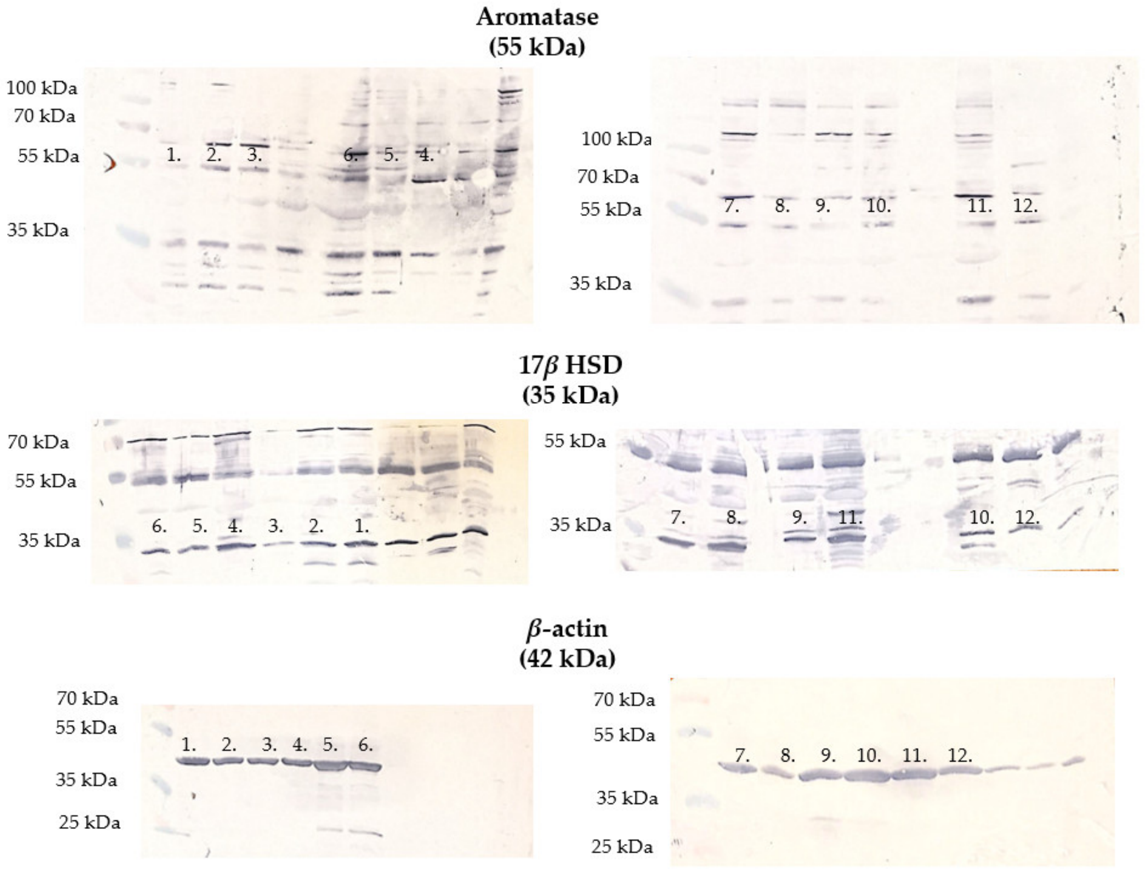

Descriptions 1. No EMF treatment, without P_4, 2 h treatment duration; **2.** 50 Hz EMF treatment, without P_4, 2 h treatment; **3.** 120 Hz EMF treatment, without P_4, 2 h treatment; **4.** No EMF treatment, with P_4, 2 h treatment duration; **5.** 50 Hz EMF treatment, with P_4, 2 h treatment; **6.** 120 Hz EMF treatment, with P_4, 2 h treatment; **7.** No EMF treatment, without P_4, 4 h treatment duration; **8.** 50 Hz EMF treatment, without P_4, 4 h treatment; **9.** 120 Hz EMF treatment, without P_4, 4 h treatment; **10.** No EMF treatment, with P_4, 4 h treatment duration; **11.** 50 Hz EMF treatment, with P_4, 4 h treatment; **12.** 120 Hz EMF treatment, with P_4, 2 h treatment.
